# A Bayesian Model for the Analysis of Transgenerational Epigenetic Variation

**DOI:** 10.1534/g3.115.016725

**Published:** 2015-01-23

**Authors:** Luis Varona, Sebastián Munilla, Elena Flavia Mouresan, Aldemar González-Rodríguez, Carlos Moreno, Juan Altarriba

**Affiliations:** * Unidad de Genética Cuantitativa y Mejora Animal, Universidad de Zaragoza, 50013, Zaragoza, Spain; † Instituto de Biocomputación y Física de los Sistemas Complejos (BIFI), Universidad de Zaragoza, 50018, Zaragoza, Spain; ‡ Departamento de Producción Animal, Facultad de Agronomía, Universidad de Buenos Aires, 1417, Ciudad Autónoma de Buenos Aires, Argentina

**Keywords:** epigenetics, Bayesian analysis, genetic variance, resemblance between relatives

## Abstract

Epigenetics has become one of the major areas of biological research. However, the degree of phenotypic variability that is explained by epigenetic processes still remains unclear. From a quantitative genetics perspective, the estimation of variance components is achieved by means of the information provided by the resemblance between relatives. In a previous study, this resemblance was described as a function of the epigenetic variance component and a reset coefficient that indicates the rate of dissipation of epigenetic marks across generations. Given these assumptions, we propose a Bayesian mixed model methodology that allows the estimation of epigenetic variance from a genealogical and phenotypic database. The methodology is based on the development of a **T** matrix of epigenetic relationships that depends on the reset coefficient. In addition, we present a simple procedure for the calculation of the inverse of this matrix (**T^−1^**) and a Gibbs sampler algorithm that obtains posterior estimates of all the unknowns in the model. The new procedure was used with two simulated data sets and with a beef cattle database. In the simulated populations, the results of the analysis provided marginal posterior distributions that included the population parameters in the regions of highest posterior density. In the case of the beef cattle dataset, the posterior estimate of transgenerational epigenetic variability was very low and a model comparison test indicated that a model that did not included it was the most plausible.

Epigenetics studies variations in gene expression that are not caused by modifications of the DNA sequence (Eccleston *et al.* 2007) and is now considered as one of the most important fields of biological research. Several biochemical mechanisms that alter gene activity (DNA methylation, histone modifications, etc.) underpin epigenetic processes (Jablonka and Raz 2009; Law and Jacobsen 2010). A number of studies have emphasized the importance of epigenetics in cancer (Esteller 2008) and other human illnesses (Feinberg 2007) as well as in other mammal (Reik 2007) and plant traits (Henderson and Jacobsen 2007). Gene activity modifications may occasionally occur in a sperm or an egg cell and can be transferred to the next generation, denoted as transgenerational epigenetic inheritance (Youngson and Whitelaw 2008), a phenomenon that has been reported in a wide range of organisms (Jablonka and Raz 2009). Despite this, the magnitude of the phenotypic variation that is explained by epigenetic processes still remains unclear (Grossniklaus *et al.* 2013; Heard and Martienssen 2014).

From the perspective of quantitative genetics, the presence of transgenerational epigenetic inheritance involves a redefinition of the covariance between relatives. Tal *et al.* (2010) developed a model for the calculation of the covariance between relatives for asexual and sexual reproduction as a function of epigenetic heritability ($\gamma^{2}$), the reset coefficient (*v*), and its complement, the epigenetic transmission coefficient (*1 − v*). According to these authors, the covariance between relatives is reduced as the number of opportunities to dissipate (or reset) the epigenetic marks increase. Therefore, for sexual diploid organisms, the covariance between parent and offspring is greater than the covariance between full sibs, and the covariance between half sibs is greater than the covariance between an uncle and its nephew, despite the fact that the additive numerator relationship between them is identical (0.5 and 0.25, respectively).

In animal and plant breeding, the standard procedure for estimating variance components is by means of a linear mixed model (Henderson 1984) that includes systematic or random environmental effects, one or more random genetic effects, and a residual. The variance components are estimated by using likelihood-based procedures (Patterson and Thompson 1971) or Bayesian approaches (Gianola and Fernando 1986). Under the Bayesian paradigm, the standard procedure for determining the posterior distribution of the variance components uses the Gibbs Sampler algorithm (Gelfand and Smith 1990) that involves an updated iterative sampling scheme from the full conditional distributions of all the unknowns in the model.

The aim of this study is to present a Bayesian linear mixed model that allows one to estimate the heritable epigenetic variability, the reset coefficient, and the epigenetic transmission coefficient based on genealogical and phenotypic information. The model is illustrated with two simulated datasets and with one example that considers a beef cattle database.

## Material and Methods

### Statistical model

The standard mixed linear model is described by the following equation:

y=Xb+Zu+e

where **y** is the vector of phenotypic records, **b** is the vector of systematic effects, **u** is the vector of random additive genetic effects, and **e** is the vector of residuals. Then, **X** and **Z** are the matrices that link the systematic and additive genetic effects with the data. The usual assumption for the prior distribution of **u** and **e** are the following multivariate Gaussian distributions (MVN):

$$u∼MVN\left(0,A\sigma_{u}^{2}\right)\;\;\;\;e∼MVN\left(0,I\sigma_{e}^{2}\right)$$

where $\sigma_{u}^{2}$ and $\sigma_{e}^{2}$ are the additive genetic and residual variances, respectively, and **A** is the numerator relationship matrix. Further, the prior distribution of **b** is commonly assumed to be a uniform distribution. Conjugate priors for the variance components are the following inverted chi-square distributions:

$$\sigma_{u}^{2}∼\chi^{-1}\left(s_{u}^{2},n_{u}\right)\;\;\;\;\sigma_{e}^{2}∼\chi^{-1}\left(s_{e}^{2},n_{e}\right)$$

where $s_{u}^{2}$ and $s_{e}^{2}$ are the prior values of the variances and $n_{u}$ and $n_{e}$ are their corresponding prior “degrees of belief”.

To estimate transgenerational epigenetic variability, this standard model can be expanded to:

y=Xb+Zu+Zw+e

where **w** is the vector of individual epigenetic effects. Note that the incidence matrix (**Z**) is the same for **u** and **w** and that both genetic and epigenetic effects are assumed to be independent. The prior distribution of **w** is defined as:

$$w∼MVN\left(0,T\sigma_{w}^{2}\right)$$

where **T** is the matrix of epigenetic relationships between individuals and $\sigma_{w}^{2}$ is the transgenerational epigenetic variance. As before, the prior distribution of $\sigma_{w}^{2}$ is defined as:

$$\sigma_{w}^{2}∼\chi^{-1}\left(s_{w}^{2},n_{w}\right)$$

with hyperparameters $s_{w}^{2}$ and $n_{w}$.

The structure of the **T** matrix is defined by the recursive relationship between the epigenetic effect of one individual ($w_{i}$) with respect to the epigenetic effects of its father ($w_{fi}$) and mother ($w_{mi}$):

$$w_{i}=\lambda w_{fi}+\lambda w_{mi}+\varepsilon_{i}$$

where

$$\lambda =\frac{1}{2}(1-v)$$

As defined by Tal *et al.* (2010), *v* is the reset coefficient and (1 − *v*) is the epigenetic transmission coefficient. The reset coefficient represents the proportion of epigenetic marks across the parental genome that are expected to be erased, whereas its opposite, the epigenetic transmission coefficient, indicates the proportion that are transmitted. Furthermore, $\varepsilon_{i}$ is the residual epigenetic effect of the *ith* individual, independent from w*_i_*_,_, and whose distribution is:

$$\varepsilon_{i}∼N\left(0,\left(1-2\lambda^{2}\right)\sigma_{w}^{2}\right)\;\text{if}\;\text{both}\;\text{parents}\;\text{are}\;\text{known};$$

$$\varepsilon_{i}∼N\left(0,\left(1-\lambda^{2}\right)\sigma_{w}^{2}\right)\;\text{if}\;\text{only}\;\text{one}\;\text{ancestor}\;\text{in}\;\text{known};$$

assuming that the variance of transgenerational epigenetics effects (V()) is constant across generations:

$$V\left(w_{i}\right)=V\left(w_{fi}\right)=V\left(w_{mi}\right)=\sigma_{w}^{2}$$

In matrix notation:

w=Pw+ε (1)

where the **P** matrix defines a recurrent relationship with the epigenetic effects of the father and mother. For nonbase individuals, the *ith* row of the **P** matrix contains a parameter λ in the column pertaining to the father and mother of the *ith* individual. The rest of elements are null.

Furthermore if:

$$w={\left[I-P\right]}^{-1}\varepsilon$$

then

$$V(w)=T\sigma_{w}^{2}={\left[I-P\right]}^{-1}V\left(\varepsilon \right){\left[I-P\prime \right]}^{-1}$$

where V(ε) is a diagonal matrix with entries equal to $\sigma_{w}^{2}$ for base individuals, $\left(1-\lambda^{2}\right)\sigma_{w}^{2}$ for individuals with one known ancestor, and $\left(1-2\lambda^{2}\right)\sigma_{w}^{2}$ for individuals whose father and mother are known. The prior distribution for λ will be assumed to be uniform, between 0 and 0.5.

### Parameter estimation through a Gibbs sampler

The Gibbs sampler algorithm is an iterative, updating sampling scheme that obtains samples from the marginal posterior distributions of all the unknowns in a model. It requires samples from the full conditional distributions of all the parameters. In the proposed model, the set of parameters can be classified into three main groups: i) the location parameters (**b**, **u**, and **w**); ii) the parameter λ associated with matrix **T**; iii) the variance components ($\sigma_{u}^{2}$, $\sigma_{w}^{2}$, and $\sigma_{e}^{2}$).

The full conditional distributions for these groups are:

#### Sampling of the location parameters (b, u, w):

The conditional distributions of the location parameters are univariate Gaussian (Wang *et al.* 1994), with parameters drawn from the following mixed model equations:

Cs=r

where

$$C=\left[\begin{array}{ccc} X^{\prime }X & X^{\prime }Z & X^{\prime }Z\\ Z^{\prime }X & Z^{\prime }Z+A^{-1}\alpha & Z^{\prime }Z\\ Z^{\prime }X & Z^{\prime }Z & Z^{\prime }Z+T^{-1}\psi \end{array}\right]\;s=\left[\begin{array}{c} b\\ u\\ w \end{array}\right]\;r=\left[\begin{array}{c} X^{\prime }y\\ Z^{\prime }y\\ Z^{\prime }y \end{array}\right]$$

and α=σe2σu2 and ψ=σe2σw2.

Specifically, the full conditional distribution for the *ith* location parameter (s_i_) is:

$$s_{i}∼N\left(\frac{r_{i}-\sum_{i\neq j}c_{ij}s_{j}}{c_{ii}},\frac{\sigma_{e}^{2}}{c_{ii}}\right)$$

given the multivariate Gaussian nature of the conditional distribution of the location parameters.

A key limitation of the procedure is the calculation of the **A^−1^** and **T^−1^** matrices. Whereas **A^−1^** is calculated by the standard Henderson’s rules (Henderson 1976) only once, the **T^−1^** matrix needs to be calculated afresh in each cycle as the parameter λ is updated. An algorithm to set-up the **T^−1^** matrix is presented in the appendix of this work (*see Appendix*).

#### The full conditional distribution of λ:

The conditional distribution of λ is developed from the recursive definition of the epigenetic effects (equation 1). It is a truncated univariate Gaussian distribution (TN) between 0 and 0.5 due to the prior distribution of the parameter.

$$p\left(\lambda |w\right)∼TN_{\left[0,0.5\right]}\left(\mu_{\lambda },\sigma_{\lambda }\right)$$

$$\mu_{\lambda }=\frac{\sum_{i=1}^{n_{1}}\frac{\left(w_{fi}+w_{mi}\right)w_{i}}{\left(\sigma_{w}^{2}\left(1-2\lambda^{2}\right)\right)}+\sum_{i=1}^{n_{2}}\frac{w_{fi}w_{i}}{\left(\sigma_{w}^{2}\left(1-\lambda^{2}\right)\right)}+\sum_{i=1}^{n_{3}}\frac{w_{mi}w_{i}}{\left(\sigma_{w}^{2}\left(1-\lambda^{2}\right)\right)}}{\sum_{i=1}^{n_{1}}\frac{{\left(w_{fi}+w_{mi}\right)}^{2}}{\left(\sigma_{w}^{2}\left(1-2\lambda^{2}\right)\right)}+\sum_{i=1}^{n_{2}}\frac{w_{fi}^{2}}{\left(\sigma_{w}^{2}\left(1-\lambda^{2}\right)\right)}+\sum_{i=1}^{n_{3}}\frac{w_{mi}^{2}}{\left(\sigma_{w}^{2}\left(1-\lambda^{2}\right)\right)}}$$

$$\sigma_{\lambda }=\frac{1}{\sum_{i=1}^{n_{2}}\frac{{\left(w_{fi}+w_{mi}\right)}^{2}}{\left(\sigma_{w}^{2}\left(1-2\lambda^{2}\right)\right)}+\sum_{i=1}^{n_{2}}\frac{w_{fi}^{2}}{\left(\sigma_{w}^{2}\left(1-\lambda^{2}\right)\right)}+\sum_{i=1}^{n_{3}}\frac{w_{mi}^{2}}{\left(\sigma_{w}^{2}\left(1-\lambda^{2}\right)\right)}}$$

where *n_1_* is the number of individuals with known fathers and mothers, *n*_2_ is the number of individuals with only father known, and *n*_3_ is the number of individuals with only mother known.

#### The full conditional distributions of the variance components ($\sigma_{u}^{2},\sigma_{w}^{2},\sigma_{e}^{2}$):

The conditional distributions for the variance components are the following inverted χ^2^ distributions

$$\begin{array}{l} \sigma_{u}^{2}∼\chi^{-2}\left(u^{\prime }A^{-1}u+s_{u}^{2},\mathrm{nan}+n_{u}\right)\\ \sigma_{w}^{2}∼\chi^{-2}\left(w^{\prime }T^{-1}w+s_{w}^{2},\mathrm{nan}+n_{w}\right)\\ \sigma_{e}^{2}∼\chi^{-2}\left(e^{\prime }e+s_{e}^{2},\mathrm{ndat}+n_{e}\right) \end{array}$$

where *nan* is the number of individuals in the population, *ndat* is the number of phenotypic data, and $s_{x}^{2}$ and $n_{x}$ are the hyperparameters for $\sigma_{x}^{2}$.

### Simulated datasets

To evaluate the procedure, we simulated two different datasets. The first one had a relative high percentage of variation caused by additive and transgenerational epigenetic effects (35% and 20%, respectively) and an intermediate reset coefficient (*v* = 0.40). In the second dataset, a lower percentage of variation was explained by additive genetic and transgenerational epigenetic effects (15% and 10%, respectively) and the reset coefficient was greater (*v* = 0.80). Each dataset was composed by a three-generation population. The base populations included 3000 individuals (1500 sires and 1500 dams) and each of the two subsequent generations were composed by 3000 full sib families of 10 individuals each one. The sire and dam for each family were sampled randomly from the individuals of the previous generation. The genetic and epigenetic effects for each individual (*u*_i_ and *w*_i_) in the base population were generated from the following Gaussian distributions:

$$\begin{array}{l} u_{i}∼N(0,\sigma_{u}^{2})\\ w_{i}∼N(0,\sigma_{w}^{2}) \end{array}$$

Furthermore, the genetic and epigenetic effects for the individuals (*u_j_* and *w_j_*) of the second and third generation were obtained by sampling from:

$$\begin{array}{l} u_{j}∼N(\frac{1}{2}u_{fj}+\frac{1}{2}u_{mj},\frac{\sigma_{u}^{2}}{2})\\ w_{j}∼N(\lambda w_{fj}+\lambda w_{mj},{\left(1-\lambda \right)}^{2}\sigma_{w}^{2}) \end{array}$$

where $u_{fi}$ and $u_{mj}$ and $w_{fi}$ and $w_{mj}$ were the additive genetic and transgenerational epigenetic effects of the father and the mother of the *jth* individual, respectively. In addition, one phenotypic record (*y_i_*) was generated for every individual by:

$$y_{i}∼N(\mu +u_{i}+w_{i},\sigma_{e}^{2})$$

where μ was a general mean set to 100 units. The values of the parameters for the two datasets were:

Dataset 1.
  $$\sigma_{u}^{2}=210,\;\sigma_{w}^{2}=120,\;\sigma_{e}^{2}=270,\;\lambda =0.30,\;h^{2}=0.35,\;\gamma^{2}=0.20,v=0.40$$

being

$$h^{2}=\sigma_{u}^{2}/\left(\sigma_{u}^{2}+\sigma_{w}^{2}+\sigma_{e}^{2}\right)\;\mathrm{and}\;\gamma^{2}=\sigma_{w}^{2}/\left(\sigma_{u}^{2}+\sigma_{w}^{2}+\sigma_{e}^{2}\right)$$

Dataset 2.
  $$\sigma_{u}^{2}=90,\;\sigma_{w}^{2}=60,\;\sigma_{e}^{2}=450,\;\lambda =0.10,\;h^{2}=0.15,\;\gamma^{2}=0.10,v=0.80$$

The Gibbs sampler was implemented using own software written in Fortran 95. The analysis consisted of a single chain of 1,250,000 cycles, and the first 250,000 were discarded. Each analysis took 36 hr using a single thread of an Intel Xeon E5-2650 of 2.00 GHz. The source code is included as Supporting Information, File S1. Convergence was checked by the visual inspection of the chains and with the test of Raftery and Lewis (1992). All samples were stored for calculating summary statistics.

### Pirenaica beef cattle data

As an illustrative example, we used a database of phenotypic records from the yield recording system of the Pirenaica beef cattle breed. The Pirenaica breed is a meat-type beef population from northern Spain with an approximate census of 20,000 individuals that are typically reared under extensive conditions (Sánchez *et al.* 2002). The data set was made up of 78,209 records for birth weight with an average value of 41.52 kg and a raw standard deviation of 4.65 kg. In addition, a pedigree file including 125,974 individual-sire-dam records was used. This information was provided by the National Pirenaica Breeders Confederation (*Confederación Nacional de Asociaciones de Ganado Pirenaico*; http://www.conaspi.net). Animal Care and Use Committee approval was not required for this study as field data were obtained from the Yield Recording System of the Pirenaica breed; furthermore, data were recorded by the stockbreeders themselves, under standard farm management, with no additional requirements.

The full model of analysis was:

$$y=Xb+Z_{1}u+Z_{2}m+Z_{2}p+Z_{3}h+Z_{1}w+e$$

Where **b** is the vector of fixed systematic effects that included sex (2 levels) and age of the mother (16 levels), **u** and **m** are the vectors of direct and maternal additive genetic effects with 125,974 elements, **p** is the vector of permanent environmental maternal effects (21,143 levels), **h** is the vector of the random herd-year-season effects (12,925 levels), **w** is the vector of transgenerational epigenetic effects (125,974 levels), and **e** is the vector of the residuals. Furthermore, **X**, **Z_1_**, **Z_2_**, and **Z_3_** are the incidence matrices that link the different effects with the phenotypic data. Appropriate uniform bounded distributions were assumed for the systematic effects and for each variance component ($\sigma_{x}^{2}=\{\sigma_{p}^{2},\sigma_{h}^{2},\sigma_{w}^{2},\sigma_{e}^{2}\}$), defined by hyperparameters (*n_x_* = −2 and *s^2^_x_* = 0). Furthermore, the prior distribution for the (co) variance matrix between direct and maternal genetic effects (**G**) was the following inverted Wishart:

$$p\left(G|n_{G},G_{0}\right)=IW(n_{G},G_{0})$$

where

$$G=\left(\begin{array}{cc} \sigma_{u}^{2} & \sigma_{um}^{}\\ \sigma_{um}^{} & \sigma_{m}^{2} \end{array}\right)$$

being $\sigma_{u}^{2}$ and $\sigma_{m}^{2}$ the direct and maternal genetic variances and $\sigma_{um}^{}$ the covariance between them. In this case, $n_{G}$ was set to −3 and **G_0_** to a 2 × 2 matrix of zeroes to define a uniform distribution.

Two statistical models (I and II) were fitted. Model I includes transgenerational epigenetic variance ($\sigma_{w}^{2}$), whereas Model II does not. For each model, the Gibbs sampler was implemented with a single chain of 3,250,000 cycles, and the first 250,000 were discarded. Convergence was checked by the visual inspection of the chains and with the test of Raftery and Lewis (1992). The models were compared based on the pseudo-log-marginal probability of the data (Gelfand 1996; Varona and Sorensen 2014) by computing the logarithm of the Conditional Predictive Ordinate (LogCPO) calculated from the Markov chain Monte Carlo (MCMC) samples. It was calculated as:

$$LogCPO=\sum_{i}\ln\overset{⌢}{p}\left(y_{i}|y_{-i},M_{k}\right)$$

where **y**_-i_ is the vector of data with the *ith* datum (y_i_) deleted, *M_k_* is the *kth* model and

$$\overset{⌢}{p}\left(y_{i}|y_{-i},M_{k}\right)=Ns{\left[\sum_{j=1}^{Ns}\frac{1}{p\left(y_{i}|\theta_{k}^{j},M_{k}\right)}\right]}^{-1}$$

being *Ns* is the number of MCMC draws, $\theta_{k}^{j}$ is the *jth* draw from the posterior distribution of the parameters of the *kth* model.

## Results and Discussion

From the perspective of quantitative genetics, the estimation of variance components is obtained from the statistical information provided by the covariance between the phenotypes of relatives (Falconer and Mckay 1996). Tal *et al.* (2010) proposed a simple model for the resemblance between relatives under transgenerational epigenetic inheritance for asexual and sexual reproduction. This model assumes that transgenerational epigenetic effects are not correlated with the additive genetic ones, and that they are distributed under a Gaussian law. After the central limit theorem, this may be explained by a large number of epigenetic marks randomly distributed across the genome. In addition, the distribution pattern of these marks is assumed independent between individuals unless they are relatives. In that case, some of these marks should have not been erased during the meiosis drawing them apart from their common ancestor. The population rate of erasure of these marks is measured through the reset coefficient (*v*).

**Figure 1:**
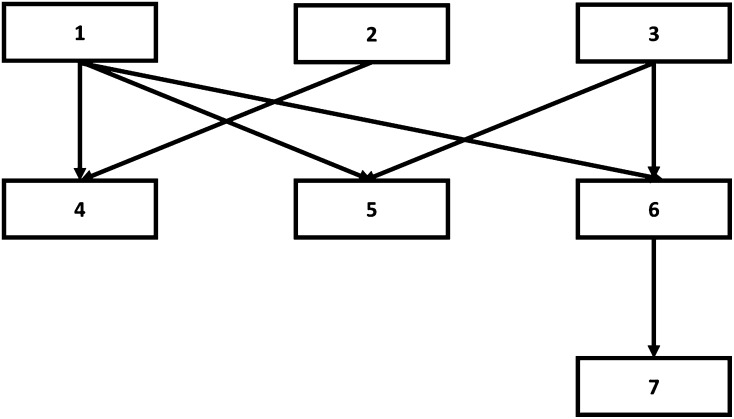
Example of a pedigree.

In this study, we propose a procedure that makes use of the aforementioned authors’ definition for sexual diploid organisms to estimate the parameters under a Bayesian mixed model framework. Our approach makes it feasible to estimate transgenerational epigenetic variability from huge datasets of genealogical and phenotypic data. The key to the procedure is the definition of a **T** matrix of (co) variance between transgenerational epigenetic effects. This matrix exclusively depends on a single parameter (λ) which is directly related to the reset coefficient (*v*) put forward by Tal *et al.* (2010). As an example, for a simple pedigree of seven individuals (Figure 1), the **T** matrix is:

$$T=\left[\begin{array}{ccccccc} 1 & 0 & 0 & \lambda & \lambda & \lambda & \lambda^{2}\\ 0 & 1 & 0 & \lambda & 0 & 0 & 0\\ 0 & 0 & 1 & 0 & \lambda & \lambda & \lambda^{2}\\ \lambda & \lambda & 0 & 1 & \lambda^{2} & \lambda^{2} & \lambda^{3}\\ \lambda & 0 & \lambda & \lambda^{2} & 1 & 2\lambda^{2} & 2\lambda^{3}\\ \lambda & 0 & \lambda & \lambda^{2} & 2\lambda^{2} & 1 & \lambda \\ \lambda^{2} & 0 & \lambda^{2} & \lambda^{3} & 2\lambda^{3} & \lambda & 1 \end{array}\right]$$

As Tal *et al.* (2010) stated, if we compare the covariance between full sibs (individuals 5 and 6) and the covariance between sire-offspring (individuals 1 and 4), the covariance is greater for the sire offspring pair, λ *vs.* $2\lambda^{2}$, as λ is defined as being within the interval (0, 0.5). Note that if the reset coefficient is 0, then λ is 1/2 and the **T** matrix becomes equivalent to the numerator relationship matrix (**A**), where the covariances between full sibs and sire offspring are equivalent. Similarly, the uncle−nephew covariance (individuals 5 and 7) is $2\lambda^{3}$, lower than the covariance between half sibs (individuals 4 and 5) which is only $\lambda^{2}$.

The mixed model implementation of the covariance between relatives requires the inverse of the **T** matrix to construct the mixed model equations, in a similar manner as the numerator relationship matrix in the standard model (Henderson 1984). In this study, we describe a simple procedure for the calculation of this **T^-1^** matrix (see the *Appendix* section). The procedure takes into account the recursive nature of the transgenerational epigenetic effects, using an argument equivalent to Quass (1976), for the inverse of numerator relationship matrix (**A^−1^**), and Quintanilla *et al.* (1999), for dam-related permanent maternal environmental effects. The main consequence is that the inverse of the **T** matrix can be sequentially constructed by reading the pedigree of the population, in the light of very simple rules. These rules are close to Henderson’s (1976) rules for constructing the inverse of the **A** matrix.

For the example pedigree, the **T^−1^** matrix is:

$$T^{-1}=\left[\begin{array}{ccccccc} 1+\frac{3\lambda^{2}}{\left(1-2\lambda^{2}\right)} & 0 & 0 & \frac{-\lambda }{\left(1-2\lambda^{2}\right)} & \frac{-\lambda }{\left(1-2\lambda^{2}\right)} & \frac{-\lambda }{\left(1-2\lambda^{2}\right)} & 0\\ 0 & 1+\frac{\lambda^{2}}{\left(1-2\lambda^{2}\right)} & 0 & \frac{-\lambda }{\left(1-2\lambda^{2}\right)} & 0 & 0 & 0\\ 0 & 0 & 1+\frac{2\lambda^{2}}{\left(1-2\lambda^{2}\right)} & 0 & \frac{-\lambda }{\left(1-2\lambda^{2}\right)} & \frac{-\lambda }{\left(1-2\lambda^{2}\right)} & 0\\ \frac{-\lambda }{\left(1-2\lambda^{2}\right)} & \frac{-\lambda }{\left(1-2\lambda^{2}\right)} & 0 & \frac{1}{\left(1-2\lambda^{2}\right)} & 0 & 0 & 0\\ \frac{-\lambda }{\left(1-2\lambda^{2}\right)} & 0 & \frac{-\lambda }{\left(1-2\lambda^{2}\right)} & 0 & \frac{1}{\left(1-2\lambda^{2}\right)} & 0 & 0\\ \frac{-\lambda }{\left(1-2\lambda^{2}\right)} & 0 & \frac{-\lambda }{\left(1-2\lambda^{2}\right)} & 0 & 0 & \frac{1}{\left(1-2\lambda^{2}\right)}+\frac{\lambda^{2}}{\left(1-\lambda^{2}\right)} & \frac{-\lambda }{\left(1-\lambda^{2}\right)}\\ 0 & 0 & 0 & 0 & 0 & \frac{-\lambda }{\left(1-\lambda^{2}\right)} & \frac{1}{\left(1-\lambda^{2}\right)} \end{array}\right]$$

Given this algorithm to set-up the **T^−1^** matrix, the implementation of a Gibbs sampling approach becomes straightforward; it merely requires sequential iterative sampling from Gaussian (**b**, **u**, and **w**), truncated Gaussian (λ) and inverted χ^2^ distributions ($\sigma_{u}^{2},\sigma_{w}^{2},\sigma_{e}^{2}$). The only minor complication is that the **T^−1^** matrix depends on parameter λ and consequently must be calculated in each Gibbs sampler’s iteration. One possible alternative to avoid this matrix inversion, although not to avoid its updating in each cycle, is to implement an approach similar to the one proposed by Rodríguez-Ramilo *et al.* (2014) for the genomic relationship matrix. However, setting up the **T** matrix is computationally more demanding than the calculation of its inverse with the algorithm proposed in this study.

**Figure 2:**
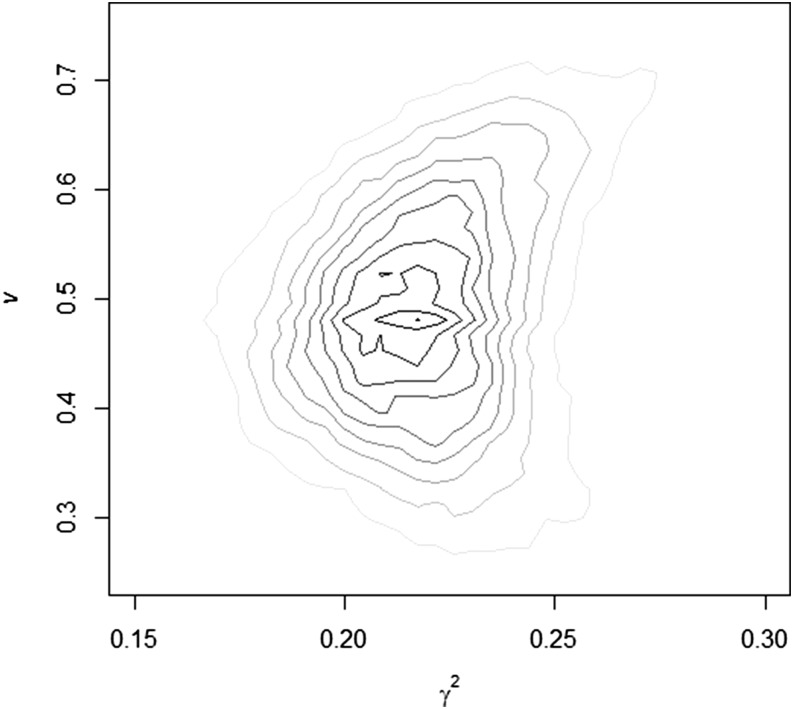
Joint posterior distribution of the transgenerational epigenetic heritability ($\gamma^{2}$) and the reset coefficient (*v*) in the first case of simulation.

**Figure 3:**
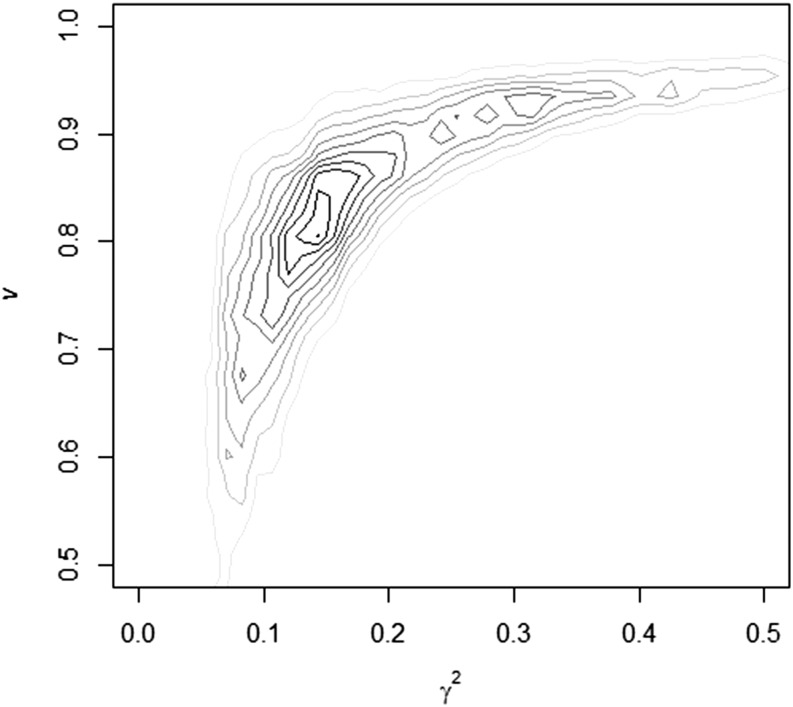
Joint posterior distribution of the transgenerational epigenetic heritability ($\gamma^{2}$) and the reset coefficient (*v*) in the second case of simulation.

The proposed procedure was first checked with simulated data. The summary of the marginal posterior distributions for the variance components, additive genetic (*h^2^*), and epigenetic ($\gamma^{2}$) heritability, reset coefficient (*v*), and epigenetic transmission coefficient (1 − *v*) for both cases of simulation are presented in Table 1. The greatest posterior density at 95% for all parameters in the model included the simulated values. However, some details of the results should be highlighted. In the second case of simulation, with lower $\sigma_{w}^{2}$ and λ, the posterior standard deviation (and the HPD95) for the $\sigma_{w}^{2}$ and $\sigma_{e}^{2}$ were remarkable wider (66.19 and 67.42, respectively). The cause of this phenomenon is that there is a statistical confounding between the epigenetic and residual variance components when the reset coefficient (*v*) is very high, because the **T** and **I** matrices becomes very similar. To illustrate this fact, in Figure 2 and Figure 3, we present the joint posterior densities of $\gamma^{2}$ and *v* for both cases of simulation. The results of the first case of simulation showed posterior independence between both parameters, whereas in the second, the marginal posterior density presented a half-moon shape. This indicates that for large values of *v*, $\gamma^{2}$ (and $\sigma_{w}^{2}$) may take any value on its support with a fairly equal probability. In other words, in the first simulated case the model showed very good ability to discriminate between both parameters, whereas in the second it did not. In addition, mixing of the MCMC procedure in the second case of simulation was clearly worst, and adaptive MCMC algorithms, such as the proposed by Mathew *et al.* (2012) may represent an interesting alternative for its implementation in large data sets.

**Table 1:** PM, PSD, and HPD95 for simulation cases I and II

	Case I			Case II		
	$\sigma_{u}^{2}=210\;\sigma_{w}^{2}=120\;\sigma_{e}^{2}=270\;\lambda =0.30$			$\sigma_{u}^{2}=90\;\sigma_{w}^{2}=60\;\sigma_{e}^{2}=450\;\lambda =0.10$		
Parameter	PM	PSD	HPD95	PM	PSD	HPD95
$\sigma_{u}^{2}$	217.87	18.21	174.66−247.49	90.91	4.63	81.42−99.25
$\sigma_{w}^{2}$	132.92	14.42	106.30−164.53	111.99	66.19	37.45−288.61
$\sigma_{e}^{2}$	251.00	19.71	205.07−282.31	396.30	67.42	217.79−475.16
$h^{2}$	0.362	0.029	0.291−0.408	0.152	0.007	0.136−0.164
$\gamma^{2}$	0.221	0.024	0.176−0.274	0.187	0.110	0.062−0.481
λ	0.256	0.055	0.148−0.365	0.091	0.059	0.020−0.233
*v*	0.488	0.111	0.270−0.704	0.818	0.117	0.534−0.960
*1 − v*	0.512	0.111	0.296−0.730	0.182	0.117	0.040−0.466

PM, posterior mean estimate; PSD, posterior standard deviation; HPD95, highest posterior density at 95%;$\sigma_{u}^{2}$, additive genetic variance, $\sigma_{w}^{2}$, transgenerational epigenetic variance;$\sigma_{e}^{2}$, residual variance. Moreover, h^2^, heritability; $\gamma^{2}$, transgenerational epigenetic heritability;λ, autorecursive parameter, *v*, the reset coefficient; 1 − *v*, epigenetic transmission coefficient.

It must be noted that the sources of information for the estimation of $\sigma_{w}^{2}$ and *v* (or λ) comes from the comparison between the covariance between different categories of relatives (see Table 2). Following Tal *et al.* (2010), one estimate of *v* can be achieved from the difference between the estimates of covariance between half-sib and uncle−nephew relationships, a difference that becomes almost null for low values of λ (or high *v*), as seen in the second case of simulation (23.10 *vs.* 22.62). On the contrary, with greater values of λ(or lower *v*), as in the first case of simulation, this difference is higher (60.60 *vs.* 57.36), and, thus, the amount of information available for estimation purposes increases. This uncertainty in the estimation of λ (or *v*) is also reflected in the estimation of $\sigma_{w}^{2}$ ($\gamma^{2}$) and $\sigma_{e}^{2}$ and implies wider posterior marginal densities. Nevertheless, it is important to emphasize that the joint posterior mode (*v* = 0.80, $\gamma^{2}$ = 0.13) in the second case of simulation was very close to the simulated parameters (0.80 and 0.10, respectively).

**Table 2:** Expected covariance between relatives in cases of simulation I and II

		Case I	Case II
Relatives	Expected Covariance	$\sigma_{u}^{2}=210\;\sigma_{w}^{2}=120\;\lambda =0.30$	$\sigma_{u}^{2}=90\;\sigma_{w}^{2}=60\;\lambda =0.10$
Offspring−Progeny	$\frac{1}{2}\sigma_{u}^{2}+\lambda \sigma_{w}^{2}$	132	51
Full-Sibs	$\frac{1}{2}\sigma_{u}^{2}+2\lambda^{2}\sigma_{w}^{2}$	121.2	46.2
Half-Sibs	$\frac{1}{4}\sigma_{u}^{2}+\lambda^{2}\sigma_{w}^{2}$	60.6	23.1
Uncle−Nephew	$\frac{1}{4}\sigma_{u}^{2}+2\lambda^{3}\sigma_{w}^{2}$	57.36	22.62

$\sigma_{u}^{2}$, additive genetic variance;$\sigma_{w}^{2}$, transgenerational epigenetic variance;λ, autorecursive parameter.

We have also checked our model with a real dataset. In Table 3, summary statistics of the marginal posterior distributions for the parameters and LogCPO values are presented for models I and II in the analysis of birth weight from the Pirenaica beef cattle population. The posterior mean estimates for heritability ranged between 0.38 (Model I) to 0.41 (Model II), and the posterior mean estimates for transgenerational epigenetic heritability was only 0.04 (Model I), with a greatest posterior density at 95% that ranged between 0.00 and 0.11. These results are coherent with the output of the model comparison test (LogCPO), which pointed to Model II as the more plausible. If we focus in this latter model, the estimates of direct and maternal heritability were within the range of estimates in the literature for these traits (Meyer 1992; Varona *et al.* 1999; Jamrozik and Miller 2014), and the absence of the epigenetic transgenerational heritability is coherent with the fact that most of the epigenetic marks are erased during the meiosis in mammals (Jablonka and Raz 2009, Schmitz 2014). However, it should be highlighted that the posterior mean estimate of the reset coefficient under Model I was surprisingly low (0.20), although its posterior standard deviation was very high (0.20), and that the HPD95 region covered almost all the parametric space (0.01−0.89). This wide range reflects the absence of information to properly estimate the parameter, given the low magnitude of $\sigma_{w}^{2}$.

**Table 3:** PM, PSD, and HPD95 for models I and II

	Model I			Model II		
	PM	PSD	HPD95	PM	PSD	HPD95
$\sigma_{u}^{2}$	9.033	0.914	7.059, 10.449	9.897	0.408	9.123, 10.727
$\sigma_{m}^{2}$	3.574	0.212	3.168, 3.998	3.554	0.217	3.135, 3.981
$\sigma_{um}^{}$	−4.473	0.253	−4.979, −3.990	−4.508	0.259	−5.032, −4.013
$\sigma_{p}^{2}$	0.747	0.081	0.590, 0.906	0.765	0.079	0.611, 0.922
$\sigma_{w}^{2}$	0.876	0.820	0.000, 2.701	−	−	−
$\sigma_{h}^{2}$	2.634	0.069	2.501, 2.771	2.633	0.069	2.499, 2.770
$\sigma_{e}^{2}$	7.055	0.223	6.606, 7.478	7.156	0.209	6.735, 7.556
λ	0.398	0.103	0.056, 0.494	−	−	−
*v*	0.204	0.207	0.012, 0.888	−	−	−
1 − *v*	0.796	0.207	0.112, 0.988	−	−	−
$h^{2}$	0.377	0.035	0.299, 0.427	0.412	0.012	0.389, 0.436
$m^{2}$	0.149	0.007	0.135, 0.164	0.148	0.007	0.133, 0.162
$\gamma^{2}$	0.036	0.034	0.000, 0.113	−	−	−
LogCPO	−287542.3			−287475.1		

PM, posterior mean estimate; PSD, posterior standard deviation; HPD95, highest posterior density at 95%;$\sigma_{u}^{2}$, additive genetic variance, $\sigma_{m}^{2}$, maternal environmental variance, $\sigma_{um}^{}$, covariance between them, $\sigma_{p}^{2}$, permanent maternal environmental variance, $\sigma_{h}^{2}$, herd-year-season variance, $\sigma_{w}^{2}$, transgenerational epigenetic variance, and $\sigma_{e}^{2}$, residual variance. Moreover, h^2^, heritability, $m^{2}$, maternal heritability, $\gamma^{2}$, transgenerational epigenetic heritability, λ, autorecursive parameter, *v*, reset coefficient, and 1 − *v*, epigenetic transmission coefficient. LogCPO, logarithm of the conditional predictive ordinate.

It is worth noting that the procedure also can provide epigenetic breeding values in the fields of animal and plant breeding. These are calculated by weighting the phenotypic information of relatives according to the magnitude of the elements of the **T** matrix. In fact, for epigenetic effects, the weight of distantly related individuals is lowered as the number of opportunities to reset the epigenetic marks increases. Both additive genetic and epigenetic effects can be used for prediction of the future performance of the individual: the expected genetic response (R) after one cycle of mass selection should be $R=i\left[h^{2}+\left(1-v\right)\gamma^{2}\right]\sigma_{p}^{2}$, where *i* is the intensity of selection and $\sigma_{p}^{2}$ is the phenotypic variance. However, further research must be undertaken to develop adequate indices of selection that consider genetic and epigenetic effects. Although both of them affect the immediate future performance of the offspring, epigenetic effects are diluted in future generations as the epigenetic marks are cleaned. It is important to note that if this selection pressure is relaxed, the average epigenetic effect declines to zero with a rate of ${\left(1-v\right)}^{n}$, where *n* is the number of generations without selection.

It should be further noted that the proposed model uses a very basic definition of transgenerational epigenetic inheritance, as it assumes equal epigenetic variance for all individuals in the population. However, when phenotypic records are recorded through the life of the individual, transgenerational epigenetic variance can be modeled as age dependent, based on the consideration that the number of epigenetic marks was accumulated in the genome of the individuals. Moreover, in the proposed model, it is assumed that the λ parameter (or the reset coefficient) is equal for fathers and mothers, and, in future research, it seems reasonable to assume a different transmission coefficient for males and females, allowing for the presence of sex differential genomic imprinting (Barlow 1997; Reik and Walker 1998). The model can be refined by the inclusion of a hierarchical Bayesian paradigm that includes systematic effects for any environmental factors that may influence the epigenetic transmission coefficient. In addition, a more profound approach could even allow for the consideration of a genetic determinism of the reset coefficient with a model similar to that proposed by Varona *et al.* (2008) for the degree of asymmetry of a skewed Gaussian distribution.

Finally, and as mentioned by Jablonka and Raz (2009) and Tal *et al.* (2010), epigenetics may be viewed as a more wide ranging concept that could include several types of cultural transmission (Avital and Jablonka 2000; Richerson and Boyd 2005). The procedure suggested in this work also could be applied to the analysis of human or animal datasets that involve any kind of transgenerational transmission, even those not directly related to gene expression.

By way of a conclusion, we can say that this paper presents an original procedure for the estimation of transgenerational epigenetic variability based on some generalized assumptions. It is hoped that the proposal will lead to future research on variations of epigenetic transmission abilities caused by environmental and/or genetic factors.

## Supplementary Material

Supporting Information

## Appendix

### Derivation of the **T^−1^** matrix

The vector of epigenetic effects (**w**) can be represented as:

w=Pw+ε

where the **P** matrix defines a recurrent relationship with the epigenetic effects of the father and mother. For nonbase individuals, the ith row of the **P** matrix contains a parameter λ in the column pertaining to the father and mother of the ith individual. The rest of the elements are null.

Furthermore, if:

$$w={\left[I-P\right]}^{-1}\varepsilon$$

then

$$V\left(w\right)={\left[I-P\right]}^{-1}V\left(\varepsilon \right){\left[I-P^{\prime }\right]}^{-1}$$

given that $V\left(w\right)=T\sigma_{w}^{2}$

$${\left[V\left(w\right)\right]}^{-1}=T^{-1}\frac{1}{\sigma_{w}^{2}}=\left[I-P\prime \right]{\left[V\left(\varepsilon \right)\right]}^{-1}\left[I-P\right]$$ (A1)

From ε=[I−P]w, it is possible to define a **Q_T_** matrix as:

$$Q_{T}=V\left(\varepsilon \right)\frac{1}{\sigma_{w}^{2}}=\left[I-P\right]T{\left[I-P\right]}^{\prime }$$ (A2)

If we consider the structure of [I−P] and $\left[I-P^{\prime }\right]$, the **Q_T_** matrix has a diagonal structure with 1s for base individuals and $1-\lambda^{2}$ and $1-2\lambda^{2}$ for those with one or two known parents, respectively.

Finally, replacing (A2) into (A1):

$$T^{-1}=\left[I-P^{\prime }\right]Q_{T}^{-1}\left[I-P\right]$$

Taking advantage of this structure, it is possible to define the following algorithm for constructing **T**^−1^ by reading the pedigree information and calculating for each individual (i) in the pedigree:

- If the father and mother are unknown;

add 1 to the diagonal (i,i)

- If only one parent (p) is known;

add $\frac{1}{\left(1-\lambda^{2}\right)}$ to the diagonal (i,i)

add $\frac{-\lambda }{\left(1-\lambda^{2}\right)}$ to the elements (i,p), (p,i)

add $\frac{\lambda^{2}}{\left(1-\lambda^{2}\right)}$ to the element (p,p)

- If both ancestors (p and m) are known;

add $\frac{1}{\left(1-2\lambda^{2}\right)}$ to the diagonal (i,i)

add $\frac{-\lambda }{\left(1-2\lambda^{2}\right)}$ to the elements (i,p), (i,m), (m,i), (p,i)

add $\frac{\lambda^{2}}{\left(1-2\lambda^{2}\right)}$ to the elements (p,p), (p,m), (m,p), (m,m)
