# Supplementary material for: A Bayesian Model for the Analysis of Transgenerational Epigenetic Variation
Source: G3 (Bethesda). 2015 Jan 23;5(4):477–85. doi: 10.1534/g3.115.016725 (PMC4390564; doi:10.1534/g3.115.016725)
Supplement: Supporting Information [file supp_5_4_477__index.html]

A Bayesian Model for the Analysis of Transgenerational Epigenetic Variation — Supporting Information 

# A Bayesian Model for the Analysis of Transgenerational Epigenetic Variation

## Supporting Information for Varona *et al.*, 2015

**Files in this Data Supplement:**

- File S1 - Computer code and datasets. (.zip, 3 MB)
